# Estimation of Foot Plantar Center of Pressure Trajectories with Low-Cost Instrumented Insoles Using an Individual-Specific Nonlinear Model

**DOI:** 10.3390/s18020421

**Published:** 2018-02-01

**Authors:** Xinyao Hu, Jun Zhao, Dongsheng Peng, Zhenglong Sun, Xingda Qu

**Affiliations:** 1 Institute of Human Factors and Ergonomics, Shenzhen University, Shenzhen 518060, China; huxinyao@szu.edu.cn (X.H.); zhaojun2016@email.szu.edu.cn (J.Z.); 2013110350@email.szu.edu.cn (D.P.); 2 Institute of Robotics and Intelligent Manufacturing, the Chinese University of Hong Kong, Shenzhen 518172, China; sunzhenglong@cuhk.edu.cn

**Keywords:** postural control, falls in the elderly, fall risk assessment, low-cost instrumented insoles, foot plantar center of pressure

## Abstract

Postural control is a complex skill based on the interaction of dynamic sensorimotor processes, and can be challenging for people with deficits in sensory functions. The foot plantar center of pressure (COP) has often been used for quantitative assessment of postural control. Previously, the foot plantar COP was mainly measured by force plates or complicated and expensive insole-based measurement systems. Although some low-cost instrumented insoles have been developed, their ability to accurately estimate the foot plantar COP trajectory was not robust. In this study, a novel individual-specific nonlinear model was proposed to estimate the foot plantar COP trajectories with an instrumented insole based on low-cost force sensitive resistors (FSRs). The model coefficients were determined by a least square error approximation algorithm. Model validation was carried out by comparing the estimated COP data with the reference data in a variety of postural control assessment tasks. We also compared our data with the COP trajectories estimated by the previously well accepted weighted mean approach. Comparing with the reference measurements, the average root mean square errors of the COP trajectories of both feet were 2.23 mm (±0.64) (left foot) and 2.72 mm (±0.83) (right foot) along the medial–lateral direction, and 9.17 mm (±1.98) (left foot) and 11.19 mm (±2.98) (right foot) along the anterior–posterior direction. The results are superior to those reported in previous relevant studies, and demonstrate that our proposed approach can be used for accurate foot plantar COP trajectory estimation. This study could provide an inexpensive solution to fall risk assessment in home settings or community healthcare center for the elderly. It has the potential to help prevent future falls in the elderly.

## 1. Introduction

Postural control refers to the control and maintenance of body’s center of mass (COM) within the base of support during static or dynamic activities [1]. It has drawn much attention from research community in recent decades. The main functional goals of postural control include postural orientation and postural equilibrium, both of which require the integration of sensory information from visual, vestibular and somatosensory systems to stabilize the body and coordinate the movement strategies [2]. Therefore, postural control is a complex skill based on the interaction of dynamic sensorimotor processes [3], and can be challenging for people with deficits in sensory functions. For example, elderly who suffered age-related degeneration in sensory systems [4,5] and people with pathological conditions (such as cerebral palsy [6], stroke [7] and Parkinson’s disease [8,9]) were found to be associated with impaired postural control.

The foot plantar center of pressure (COP) has often been used for quantitative assessment of postural control. For example, Rocchi et al. examined 14 COP measures by using the principle component analysis and found that five of these measures, including root mean square distance, mean velocity, principal sway direction, centroidal frequency of the power spectrum, and frequency dispersion, can effectively reflect the postural control mechanism among patients with Parkinson’s disease [9]. Liu et al. used the velocity of COP trajectory during quiet upright standing to quantify the intensity of postural sway among young adults, healthy old adults and fall prone old adults [10]. Lafond et al. suggested that the velocity of COP trajectory was the most reliable measure for assessing postural stiffness among the elderly [11]. Biswas et al. have shown that the characteristics of COP can enhance the predictive power to a constructed index for dynamic stability [12]. In other studies, COP measures were also used to assess postural stability among stroke patients [13], patients with post-stroke hemiparesis [14] and patients with rheumatic disease [15]. In a recent study, Johansson et al. investigated how the COP sway can be used to predict future falls. They found that fall risk increased by 75% for participants with the COP sway lengths ≥ 400 mm during quiet standing with eyes open. They also suggested that fall risk could almost be doubled if the sway lengths ≥ 920 mm during quiet standing with eyes closed [16].

Conventionally, the COP trajectory is measured by force plates or force mapping systems [17]. However, such systems are restricted in laboratory settings. As such, they cannot be used to assess postural control in daily activities. To address this, a variety of insole-based plantar pressure measurement systems have been developed. Some of them are commercially available, such as the F-scan measurement system (Tekscan, Inc., Boston, MA, USA) and Novel Pedar system (Novel Inc., Kirkland, WA, USA). These systems allow the COP trajectory to be captured in a more extended space compared to force plates and force mapping systems. However, they have to rely on cables for data acquisition and power supply, which makes them obstructive and compromise their wearability. More importantly, these systems are too expensive for personal or daily use in home settings.

Providing inexpensive and wearable solutions for postural control assessment will provide vital information of fall risks, and thus could be useful for preventing future falls especially among elderly [18]. Recent advancement in microelectronics technology makes such wearable solutions feasible. For instance, Balaga et al. proposed a method that used a single body-fixed inertial sensor to quantitatively describe the postural control strategy during the lying-to-sit-to-stand-to-walk transfer tasks [19]. Similarly, wearable sensors (including those integrated in smart phones) were reported to be useful for fall risk assessment [20,21,22,23]. Many low-cost instrumented insole systems have also been developed [24,25,26,27,28,29,30,31,32,33]. Among the existing low-cost insole systems, different sensor technologies were implemented, such as force sensitive resistors (FSRs) [25,33], fabric or textile pressure sensing arrays [28], or piezoelectric sensors [32], etc. The numbers of pressure sensors or sensing arrays also varied among different studies, ranging from four FSRs [31] to 48 pressure sensor arrays [32].

Regardless of the sensor types and the number of sensors, the “weighted-mean approach” was most commonly used to estimate COP trajectories. In the weighted-mean approach, the force/pressure detected by each sensor was weighted by its corresponding coordinate (i.e., sensor location) and then summed. The COP trajectory was calculated by dividing the sum of weighted force/pressure by the overall force/pressure [26,27,28,29,30,32,33]. The accuracy of the weighted-mean COP trajectory estimation approach is not robust. In particular, when implementing the weighted-mean approach, the accuracy of COP trajectory estimation is dependent on the areas covered by the pressure sensors, which will be a potential source for errors. In other words, the weighted-mean approach depends on the locations and numbers of the pressure sensors. Theoretically, more sensors or sensor arrays implemented in the insole will result in higher accuracy. However, more sensors or sensor arrays always means high cost and system complexity, which makes it practically infeasible today.

In the present study, we aimed to develop and evaluate low-cost instrumented insoles for the estimation of foot plantar COP trajectories. In order to address the limitations of the weighted-mean approach, we proposed a novel individual-specific nonlinear model. This model was expected to accurately estimate foot plantar COP trajectories using the data from a small number of low-cost FSR sensors. An experiment involving a variety of postural control assessment tasks was carried out to provide data for least square error approximation and model coefficients specification. The accuracy of the foot plantar COP trajectories estimation by this model was examined by comparing the estimated COP data with the reference data from a commercial insole-based plantar pressure measurement system. Accuracy comparisons were also carried out between the proposed nonlinear model and the weighted-mean approach. 

## 2. Materials and Methods 

### 2.1. Hardware Design

**Figure 1:**
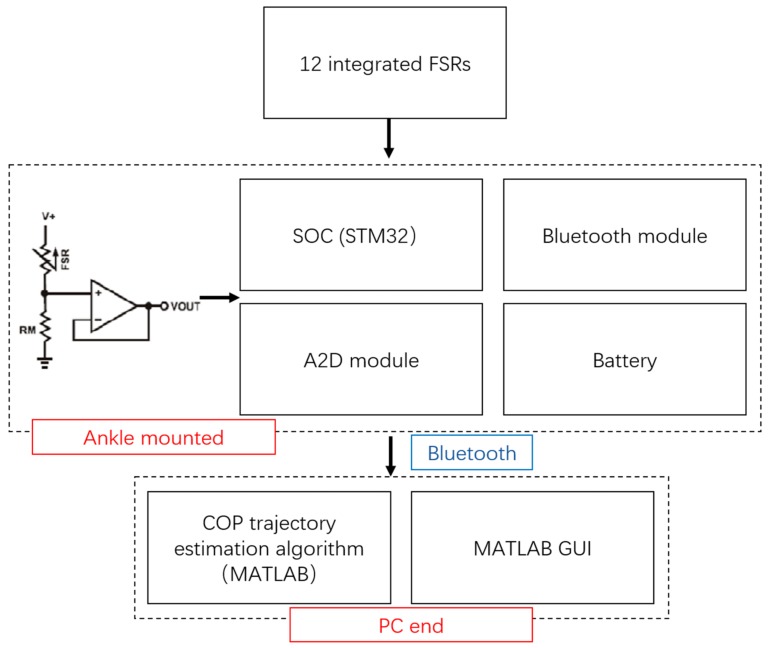
The block diagram of the instrumented insole.

**Figure 2:**
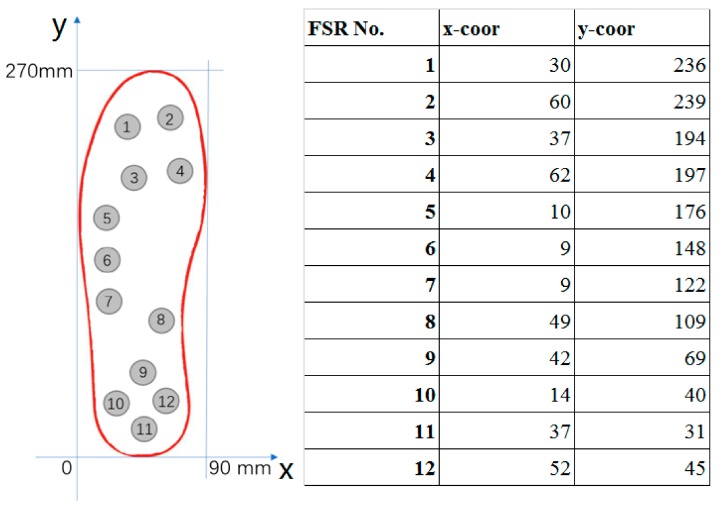
The layout of the 12 Force Sensitive Resistor (FSR) sensors and the corresponding coordinates.

The block diagram of the proposed instrumented insole is shown in Figure 1. It mainly consists of three parts: a 12 integrated FSR (FSR402, Interlink Electronics, Los Angeles, CA, USA) based insole, a lower-shank mounter block, and a PC end graphic user interface (GUI). Each FSR402 sensor has a 12.7 mm diameter sensing area made of fiberglass resin that is attached to a base 18.1 mm in diameter. The lower-shank mounter block consisted of a Micro-Computer Unit (MCU, STM32 32-bit ARM Cortex, ARM, Ltd., Cambridge, UK), a Bluetooth module (HC-06, Wavesen Co. Ltd. Guangzhou, China), a customized A2D module and a battery. Twelve FSRs were strategically adhered onto a silicone made insole, the size of which was US 9. The layout of the FSRs is depicted in Figure 2. This layout is similar to what has been suggested by Howell et al. [34], where the 12 sensors can cover the important foot plantar pressure distribution areas such as great toe, metatarsophalangeal joint, arch of the foot, and heel. An insole coordinate system was used to help identify the actual location of each sensor. As illustrated in Figure 2, the *x*-axis is the tangent line to the bottom edge of the insole, which defines the medial–lateral direction (ML); and the *y*-axis is the tangent line to the left edge of the insole, which defines the anterior–posterior (AP) direction. The origin is the intersection between the *x*-axis and the *y*-axis. 

Prior to usage, each FSR was calibrated and conditioned following the techniques suggested by Hall et al. [35]. This was to eliminate the creep effect (i.e., the change of FSR resistance over prolonged time) and minimize the hysteresis effect. After that, each FSR sensor was connected to a Voltage-to-Current (V2C) converting circuit, as recommended by the manufacturer [36]. The thickness of the connecting cable is only 0.4 mm so that it will not lead to uneven surface of the insole. The V2C converting circuit converts the FSR resistance value to an inverse voltage output, which were subsequently converted into readable voltage output through a 10-bit analog-2-digital (A2D) module. The force measured by each sensor was obtained based on its voltage output following a fourth order polynomial equation, as suggested by Hall et al. [35]. The data were transmitted wirelessly by a Bluetooth module to a PC end, where the GUI was designed by a customized MATLAB script to visualize the pressure output in real time. 

### 2.2. The Experiment

The experiment was carried out for model development and validation. A convenience sample of 10 young (age = 22.6 ± 1.5 years, height = 173.8 ± 4.4 cm, weight = 64.9 ± 4.4 kg) and 10 older adults (age = 65.7 ± 3.4 years, height = 167.5 ± 3.9 cm, weight = 65.2 ± 2.9 kg) were recruited from the local community. These participants all had the shoe size of US 9. The young participants were between 21 and 24 years old. The elderly participants were all above 60 years old and living independently, and could perform the activities of daily living (ADLs) without external assistance. Both young and elderly participants reported no injuries, illness, or medical surgery history. All participants signed the informed consent form approved by the Shenzhen University ethics committee.

**Figure 3:**
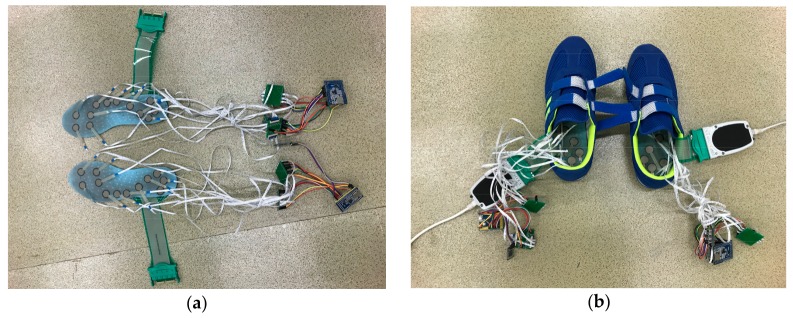
(**a**) the F-scan sensor sheets (green) were tailored and adhered underneath the instrumented insole (blue); (**b**) the insoles were inserted into a pair of sports shoes.

Previous research has demonstrated that the F-scan system can measure the COP trajectory accurately [37]. Therefore, the foot COP trajectories obtained from the F-scan system were considered as the reference measurement (i.e., the response data). Prior to the experiment, the F-scan sensor sheets were tailored to make sure it had the same shape and size as the instrumented insoles. They were then pasted underneath the instrumented insoles (Figure 3a). Cautious actions were taken before data collection to make sure that the instrumented insoles and F-scan sensor sheet were adhered evenly and firmly. The insoles were inserted into a pair of sports shoes (size US 9), as depicted in Figure 3b. 

**Figure 4:**
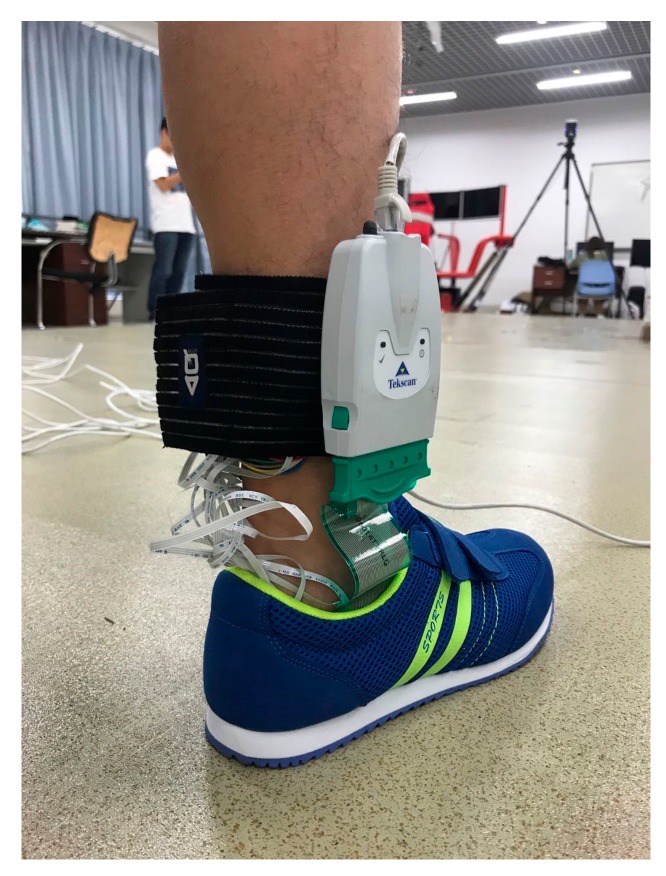
Both the lower-shank mounted block of the instrumented insole and the signal box of the F-scan system were attached to the lower-shank.

Participants were asked to wear the shoes with the instrumented insoles. Both the lower-shank mounted block of the instrumented insoles and the signal box of the F-scan system were attached to the lower shank (Figure 4). Then, participants were instructed to perform a variety of postural control assessment tasks including (1) quiet standing with open eyes, (2) quiet standing with closed eyes, (3) standing up from a chair with armrests (wooden chair, seat height 435 mm, armrests height 250 mm), (4) sitting down to a chair with armrests, (5) standing up from a chair without armrests (wooden chair, seat height 435 mm), and (6) sitting down to a chair without armrests. During quiet standing trials, participants were asked to stand quietly with a self-chosen comfortable posture for 10 s. During standing up and sitting down trials, participants were asked to keep the feet on the ground when they started standing up or sitting down. When performing standing up and sitting down tasks with the chair with armrests, participants were instructed to use the armrests to help the body ascending and descending. These tasks were performed in a random order. Three trials were collected for each task. Each of these trials was separated to form three blocks of trials, which would be used for cross validation purposes. Prior to data collection, participants were given approximately 5 min practice to get familiar with these tasks. The activation of the F-Scan system and our proposed instrumented insoles is initiated with the application of external pressure. Thus, these two insole systems were synchronized by manually applying external pressure onto them at the same time. The sampling frequency was set at 50 Hz.

We noted that the instrumented insole was placed in between the foot and F-scan sensor sheet. The overlapping between the instrumented insole and F-scan sensor sheet might modify the foot plantar pressure distribution and induce errors in F-scan measurement. Such errors might compromise the accuracy of the reference data. A test was carried out to examine the possible errors caused by the overlap. One male volunteer (age: 31, height: 180 cm, weight: 65 kg, shoe size: US 9) participated. Firstly, the participant was asked to wear the experimental shoes that had both the proposed instrumented insole and F-scan sensor sheet (overlapping condition), and perform the above-mentioned postural control assessment tasks. Each task was performed 20 times in a random order. Then, the participant wore the same shoes with F-scan sensor sheet only (no overlapping condition) and performed each postural control assessment task 20 times in a random order. Two COP parameters including COP range and mean COP were compared between the ‘overlapping’ and ‘no overlapping’ conditions by using *t*-tests. As shown in Table 1, when the level of significance was set at 0.05, no significant differences existed in the COP parameters between the ‘overlapping’ and ‘no overlapping’ conditions. This suggests that the possible errors due to the overlapping did not significantly affect the COP estimation results. Thus, we used the F-scan measurement data in the overlapping condition as the reference data in the following analysis. 

### 2.3. The Individual-Specific Nonlinear Model for COP Estimation

The foot plantar COP is practically defined as the centroid of all the external forces acting on the plantar surface of the foot [38]. Therefore, in the previous weighted-mean approach, the COP trajectories were estimated by a spatial average of all the forces measured by pressure sensors. However, with a reduced number of pressure sensors, the external forces acting on the foot plantar might not be registered completely. Thus, errors might be induced. 

To address the limitation of the weighted-mean approach, a multivariate nonlinear regression model was proposed where the FSR sensor outputs were considered as the predictor data. Similar to the weighted-mean approach, in the proposed model, the output of FSR sensor was weighted and summed to determine COP locations (Equations (1) and (2)). However, instead of using the sensor locations, the model coefficients (i.e., weighting parameters) were determined by a least square error approximation process. The sensor locations (i.e., the coordination of each sensor defined in Figure 2) were only used to set the initial values of the model coefficients. The least square error approximation process would tune the model coefficients to achieve improved COP trajectory estimation capability. This model is as follows:

(1) $$X_{COP}=\frac{\sum_{i}^{12}C_{i}^{x}F_{i}}{C_{tot}^{x}F_{tot}},$$

(2) $$Y_{COP}=\frac{\sum_{i}^{12}C_{i}^{y}F_{i}}{C_{tot}^{y}F_{tot}},$$

where $X_{COP}$ and $Y_{COP}$ are the COP coordinates defined in the coordinate system (Section 2.1). $X_{COP}$ represents the COP location along the medial–lateral direction, $Y_{COP}$ represents the COP location along the anterior–posterior direction. $C_{i}^{x}$, and $C_{i}^{y}$ (*i* = 1, 2, 3…12) are the model coefficients that weight each sensor output ($F_{i}$). $C_{tot}^{x}$ and $C_{tot}^{y}$ are the model coefficients that weights the sum of all forces registered by FSRs. $F_{tot}$ is the sum of all forces registered by FSRs ($F_{tot}=\overset{12}{\underset{i}{\sum }}F_{i}$). $C_{i}^{x}$, $C_{i}^{y},$ $C_{tot}^{x}$ and $C_{tot}^{y}$ are determined through the least square error approximation process.

An iterative least square error approach was carried out to determine the model coefficients. The model coefficients were written in the vector form as:

(3) $$\hat{C^{x}}=\left(C_{i}^{x};C_{tot}^{x}\right),$$

(4) $$\hat{C^{y}}=\left(C_{i}^{y};C_{tot}^{y}\right),$$

The reference measurement (i.e., the foot COP trajectories obtained from the F-scan system) in the vector form was as follows:

(5) $$X_{COP}=\left(X_{COP,1};X_{COP,2};\ldots X_{COP,m}\right),$$

(6) $$Y_{COP}=\left(Y_{COP,1};Y_{COP,2};\ldots Y_{COP,m}\right),$$

where m is the number of observations in the training data (which related to the time taken in each postural control assessment task). Similarly, the predictor data (F) can be written as a m×i matrix (i is the number of FSR sensors) as follows:

(7) $$F=\left(\begin{array}{ccccc} F_{11} & F_{12} & \cdots & F_{1i} & \overset{12}{\underset{i}{\sum }}F_{1i}\\ F_{21} & F_{22} & \cdots & F_{2i} & \overset{12}{\underset{i}{\sum }}F_{2i}\\ \vdots & \vdots & \ddots & \vdots & \vdots \\ F_{m1} & F_{m2} & \cdots & F_{mi} & \overset{12}{\underset{i}{\sum }}F_{mi} \end{array}\right)=\left(\begin{array}{cc} F_{1}^{T} & \overset{12}{\underset{i}{\sum }}F_{1}\\ F_{2}^{T} & \overset{12}{\underset{i}{\sum }}F_{2}\\ \vdots & \vdots \\ F_{m}^{T} & \overset{12}{\underset{i}{\sum }}F_{m} \end{array}\right).$$

The goal of the approximation process is to find $\hat{C^{x}}$ and $\hat{C^{y}}$ that can minimize the square error between the reference measurements and the model predicted values. Let $E_{\hat{C^{x}}}={\left(X_{COP}-F\hat{C^{x}}\right)}^{T}\left(X_{COP}-F\hat{C^{x}}\right)$ and $E_{\hat{C^{y}}}={\left(X_{COP}-F\hat{C^{y}}\right)}^{T}\left(X_{COP}-F\hat{C^{y}}\right)$, so

(8) $${\hat{C^{x}}}^{*}=\underset{\hat{C^{x}}}{\text{arg min}}E_{\hat{C^{x}}},$$

(9) $${\hat{C^{y}}}^{*}=\underset{\hat{C^{y}}}{\text{arg min}}E_{\hat{C^{y}}}.$$

In multivariable calculus, to find the minimum value of $E_{\hat{C^{x}}}$ and $E_{\hat{C^{y}}}$, it requires solving the following partial derivative functions [39]. This process went iteratively until the closed form optimal solution for $\hat{C^{x}}$ and $\hat{C^{y}}$ were found:

(10) $$\frac{\partial E_{\hat{C^{x}}}}{\partial \hat{C^{x}}}=2F^{T}\left(F\hat{C^{x}}-X_{COP}\right)=0,$$

(11) $$\frac{\partial E_{\hat{C^{y}}}}{\partial \hat{C^{y}}}=2F^{T}\left(F\hat{C^{y}}-Y_{COP}\right)=0.$$

Three-fold cross validation was implemented for model evaluation. The data from the postural control assessment tasks were equally assigned into three groups, and each group contained data from tasks (1)–(6) (as described in Section 2.2). For each evaluation procedure, two groups were selected as the training set while the other group as the evaluation set. This repeated three times until all the group combinations were tested. Then, the mean value of root mean square errors (RMSE), the correlation coefficients (CC), the maximum error (MaxE) and minimum error (MinE) between the estimated COP trajectories and the reference measurements were calculated. In addition, in order to determine the statistical significance of the CC, the *p*-values were examined for CC. In addition, the COP trajectory was also estimated by the weighted mean approach. Similarly, three-fold cross validation was implemented to evaluate the weighted mean approach. Comparisons were carried out between the nonlinear model and weighted mean approach in RMSE, CCs, MaxE and MinE.

### 2.4. Graphic User Interface (GUI)

**Figure 5:**
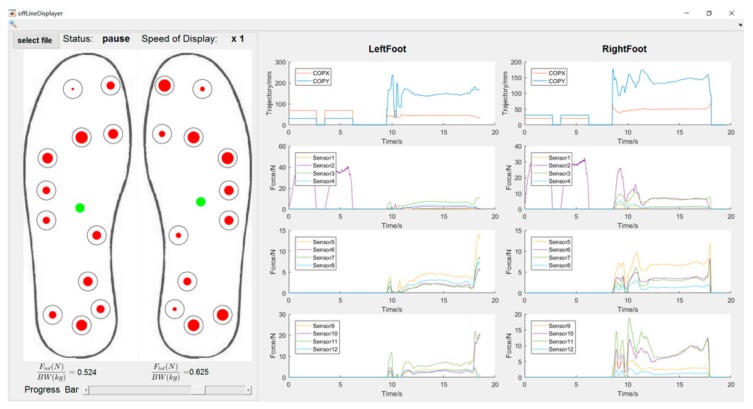
The GUI developed in the MATLAB to show the output of each sensor, $F_{tot}$ normalized by body weight (BW), and the estimated foot plantar COP trajectory.

The GUI is shown in Figure 5. The left-hand side panel shows the pair of instrumented insole and the locations of FSR sensors. Each red dot presents the instant sensor pressure, the size of which corresponds to the pressure magnitude. The green dot indicates the estimated location of the COP. The right-hand side panel shows the COP trajectories along the time. It also shows the pressure output of each FSR sensor and instant $F_{tot}$ normalized by body weight (BW).

## 3. Results

**Figure 6:**
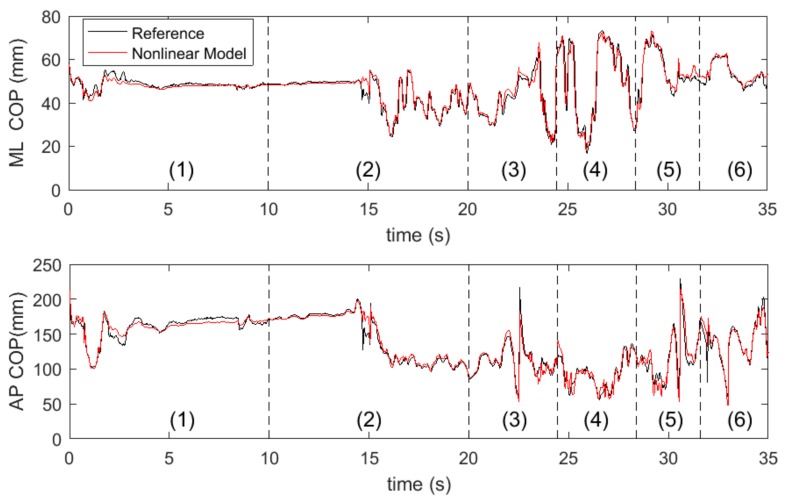
Representative plot of the comparison of COP trajectory estimation by the nonlinear model and the reference data. The data were obtained from the left foot and for the different tasks: (1) quiet standing with open eyes, (2) quiet standing with closed eyes, (3) standing up from a chair with armrests, (4) sitting down to a chair with armrests (task 3 and 4 have multi-contacts phases where participant’s hands are in contact with armrests or the seat) (5) standing up from a chair without armrests, and (6) sitting down to a chair without armrests.

Figure 6 shows an example of COP trajectory estimation by the proposed nonlinear model. The COP trajectories along the ML direction and AP direction were plotted against the reference measurements. This example shows that the estimated COP trajectories were trending closely with the reference, suggesting an accurate estimation. 

Table 2 shows the RMSE, CC, MaxE and MinE results of the COP trajectories calculated by the proposed nonlinear model compared with the reference measurements. The overall mean and standard deviation were also summarized. The RMSE reflects estimation accuracy. For the ML COP, the mean RMSEs were 2.23 (±0.64) and 2.72 (±0.83) for the left and right foot, respectively. For the AP COP, the mean RMSEs were 9.17 (±1.98) and 11.19 (±2.98), respectively. The RMSEs along the AP were larger than that along the ML direction because the COP trajectories typically have a larger moving range along the AP direction. The small RMSEs suggested that the proposed COP trajectory estimation model had high COP trajectory estimation accuracy. 

The CC accounts for the similarity of the estimated COP trajectory time series, compared to the reference measurements. The mean value of CCs was 0.91 (±0.05) to 0.93 (±0.02) along the ML and AP direction, respectively. In addition, the *p*-values of each CC were all smaller than 0.0001, indicating that the correlation is highly significant. 

The MaxE and MinE indicate the least and best estimation each model can achieve. The MaxE for ML COP was 26.119 mm and 25.254 mm for the left and right foot, respectively; for AP COP, it was 124.86 mm and 116.18 mm for the left and right foot, respectively. As for the minimum error, both the weighted mean approach and nonlinear model can achieve a high accuracy with the minimum error less than 1 mm. 

**Figure 7:**
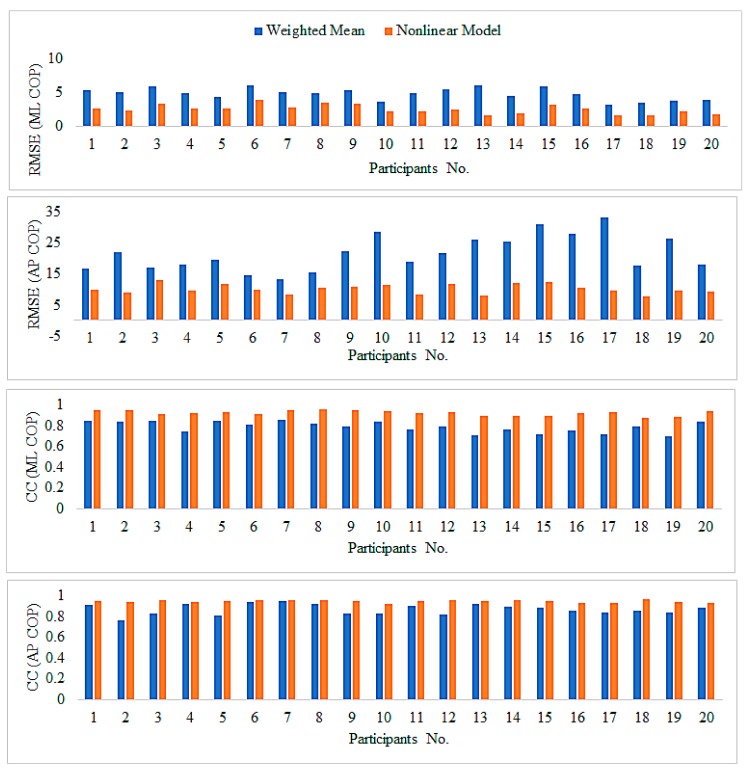
Comparisons of the RMSE and CC between the nonlinear model and the weighted mean approach.

Figure 7 shows the comparison of the RMSE between the nonlinear model and weighted mean approach. For all participants, the RMSE between the estimated COP by the nonlinear model and the reference measurements are smaller compared to their counterparts estimated by the weighted mean approach. Figure 7 also shows the CCs. Overall, the weighted mean approach yielded a fairly good CC, which was approximately 0.77–0.85 along the ML and AP direction, respectively. However, the nonlinear model yielded a higher CC. These results indicate that the COP trajectories estimated by the nonlinear model have a more similar trending with the reference COP. 

The least square error approximations were completed by a customized MATLAB script running on a laptop computer (Intel^®^ Core (TM) i7-7500U 2.7GHz, 8.00GB RAM, 256GB HD, Windows x64 operation system, HP Inc., Palo Alto, CA, USA). The number of iterations in least square error approximation was between 5–8 (Mean ± SD = 6.5 ± 1.0 iterations) across different trials. It took 0.053–0.124 s (Mean ± SD = 0.112 ± 0.005 s) to complete the approximation procedure.

## 4. Discussion

This study presents an individual-specific nonlinear model that can help estimate the foot plantar COP trajectories with an instrumented insole. Among the 20 participants involved in this study, the average RMSE between the estimated COP trajectories by the proposed model and the reference measurements was less than 3 mm along the medial–lateral direction of the foot, and less than 12 mm along the anterior–posterior direction. In addition, the estimated COP trajectories by this nonlinear model established high correlation coefficients with the reference measurements (0.91–0.93 with *p*-values all less than 0.0001). To our knowledge, the results are the smallest errors and highest correlation coefficients reported in relevant studies where the foot plantar COP trajectories were estimated by a small number of low-cost pressure sensors [28,29]. For instance, Shu [28] reported a mean relative difference of 7.6 and 9.9 mm between the estimated COP trajectories and the reference measurements during normal standing and standing on one leg. Dyer et al. [29] reported the RMSE ranging from 7 mm to 24 mm during locomotion. Overall, the results suggested that the proposed nonlinear model had excellent COP trajectory estimation capability. 

Previously, the weighted mean approach was the most commonly used method to estimate the foot plantar COP trajectory by instrumented insoles [40]. However, as mentioned earlier, this approach may not lead to desirable estimation, especially when the number of low-cost pressure sensors is small. To address the limitation of the weighted mean approach, we proposed the nonlinear model here and used this model to estimate COP trajectories during a variety of postural control assessment tasks. Meanwhile, to identify the improvement of the proposed nonlinear model, the weighted mean approach was also used for COP trajectory estimation by using the same set of data. The results show that the mean RMSE between the estimated COP trajectories by the weighted mean approach and the reference measurements were ~4–6 mm along the ML direction, and ~18–20 mm along the AP direction. These nearly doubled the RMSEs obtained by the nonlinear model. The correlation coefficients were ranging from 0.80 to 0.86, which were also lower than those calculated by the nonlinear model approach. These results suggested that the proposed nonlinear model can lead to improved COP trajectory estimation compared to the weighted mean approach.

There might be two reasons that can help explain why the nonlinear model works better than the weighted mean approach with a small number of sensors. First, in the weighted mean approach, the accuracy depends largely on the numbers of sensors and their predefined locations. As we discussed earlier, the small number of sensors might induce errors. Different sensor placement strategy will also influence the accuracy of COP trajectory estimation. However, in the nonlinear model, least square error approximation will help rectify the errors due to the small number of sensors and their placement strategy. Another reason is that the weighted mean approach cannot address individual differences, which may induce additional intra-subject errors. In addition, the least square error approximation process in the nonlinear model is dependent on individual data from the sensors. In other words, the model coefficients were determined by each participant’s own experimental data. Thus, the proposed nonlinear model is individual-specific and is able to address individual differences. 

This study demonstrated that the foot plantar COP can be accurately estimated by a small number of low-cost pressure sensors. Many instrumented insole systems have been developed and are even commercially available. Clinically, more accurate COP estimation help better postural control assessment. However, there is always a trade-off between the number of sensors and estimation accuracy. The increased number of sensors will complicate the instrumented insole system and possibly compromise the reliability of the whole system. In addition, a larger number of sensors or sensor array will increase the cost substantially and make it unaffordable for the home-dwelling elderly. Therefore, an instrumented insole with a small number of low-cost sensors like what we proposed in the present study has its own merits. 

From a practical point of view, this study can benefit the home-based postural control assessment for elderly and patients with pathological conditions. The impaired postural control is considered as an important risk factor of falls [41,42]. Though consensus is still lacking, postural control parameters have been widely suggested as indicators of fall risks [43,44,45,46]. Based on these, the authors believe that this can benefit the fall prevention research. 

There are still some limitations in this study. First, this study mainly focused on postural control assessment during static stance and sit-to-stand transitions. Although previous research suggested that increased postural sway during stance can be a risk factor for prospective falls in community-dwelling elderly individuals [10], future work needs to be carried out to test the validity of this nonlinear approach during other activities of daily living, such as walking and stair negotiation. Second, we did not examine insole size other than US 9 just for convenience. However, as the proposed COP estimation model in the present study was individual-specific, we believe that this model would be applicable for other insole sizes as well. 

## 5. Conclusions

Falls are still a major safety and health problem among aged population. Fall risk assessment is an effective approach to reduce fall accidents among the elderly. A substantial number of falls in the elderly result from loss of balance. Thus, the plantar COP, as an indicator of postural control performance, is an important fall risk assessment parameter. This study presented a low-cost instrumented insole system that uses a nonlinear model for COP trajectory estimation. Results show that this system is able to provide accurate COP trajectory data. Compared to traditional COP trajectory estimation approaches (i.e., weighted mean approach), the proposed nonlinear model performed better in terms of estimation accuracy. Based on this, we suggest that the proposed instrumented insole system could serve as an inexpensive solution to fall risk assessment in home settings or community healthcare centers for the elderly. It has the potential to help prevent future falls in the elderly.

## Figures and Tables

**Table 1:** Comparisons of COP parameters between the ‘overlapping’ and ‘no overlapping’ conditions.

		Medial-Lateral (ML) COP			Anterior-Posterior (AP) COP		
		Overlapping	No Overlapping	*p*-Value	Overlapping	No Overlapping	*p*-Value
Task 1	COP range (mm)	2.5 ± 0.3	3.5 ± 0.2	0.83	6.9 ± 2.5	5.4 ± 2.1	0.54
	Mean COP (mm)	42.4 ± 1.4	46.3 ± 3.2	0.19	189.8 ± 2.6	192.3 ± 2.3	0.73
Task 2	COP range (mm)	8.7 ± 1.2	7.7 ± 1.3	0.55	16.4 ± 3.0	13.0 ± 3.5	0.55
	Mean COP (mm)	45.2 ± 7.2	47.9 ± 6.4	0.18	193.9 ± 1.6	196.7 ± 2.4	0.18
Task 3	COP range (mm)	15.4 ± 1.7	12.5 ± 1.9	0.89	67.0 ± 18.3	68.6 ± 22.1	0.81
	Mean COP (mm)	45.7 ± 0.7	50.1 ± 1.5	0.30	187.3 ± 18.8	186.9 ± 14.3	0.91
Task 4	COP range (mm)	16.3 ± 1.9	14.5 ± 2.2	0.74	70.4 ± 15.3	74.6 ± 19.2	0.66
	Mean COP (mm)	48.4 ± 3.5	49.0 ± 4.7	0.25	186.9 ± 13.8	183.7 ± 15.5	0.96
Task 5	COP range (mm)	13.4 ± 3.3	14.9 ± 4.5	0.12	73.2 ± 11.5	70.0 ± 14.7	0.81
	Mean COP (mm)	45.1 ± 5.7	48.7 ± 3.1	0.06	208.7 ± 29.2	200.7 ± 22.1	0.78
Task 6	COP range (mm)	12.8 ± 1.5	15.1 ± 2.0	0.83	70.6 ± 10.9	74.3 ± 11.8	0.86
	Mean COP (mm)	47.5 ± 0.8	43.9 ± 0.9	0.13	204.8 ± 9.5	213.2 ± 9.8	0.62

**Table 2:** The RMSE (Root Mean Square Error), Correlation Coefficient (CC), Maximum Error (MaxE) and the Minimum Error (MinE) of the COP trajectory estimated by the nonlinear mode and the reference measurement, for both feet (L_ and R_), and along the medial-lateral (ML) and anterior-posterior (AP) directions.

Participants	RMSE (mm)				CC				MaxE (mm)				MinE (mm)			
	Left AP	Left ML	Right AP	Right ML	Left AP	Left ML	Right AP	Right ML	Left AP	Left ML	Right AP	Right ML	Left AP	Left ML	Right AP	Right ML
1	5.9	2.4	4.7	2.8	0.93	0.97	0.76	0.93	15.8	6.7	16.1	11.4	0.0	0.1	0.0	0.0
2	6.0	1.7	4.0	3.0	0.82	0.93	0.85	0.97	15.4	8.8	15.6	7.7	0.0	0.1	0.0	0.0
3	4.9	3.0	6.9	3.5	0.85	0.92	0.83	0.91	18.5	6.7	24.0	13.8	0.1	0.0	0.1	0.1
4	6.2	2.6	3.6	2.6	0.64	0.91	0.85	0.93	16.4	6.0	20.3	11.7	0.1	0.1	0.1	0.0
5	3.8	2.7	4.6	2.6	0.76	0.90	0.93	0.96	15.8	10.3	14.8	12.5	0.0	0.0	0.0	0.1
6	4.4	4.0	7.6	3.8	0.71	0.86	0.91	0.95	21.1	12.5	21.7	12.6	0.0	0.0	0.0	0.1
7	3.8	2.2	6.1	3.2	0.96	0.97	0.75	0.93	14.8	9.8	18.1	11.9	0.0	0.0	0.0	0.1
8	3.9	2.2	5.8	4.6	0.90	0.98	0.74	0.93	14.7	7.8	23.5	7.7	0.0	0.1	0.1	0.0
9	4.0	2.5	6.5	4.2	0.91	0.97	0.67	0.93	13.0	8.3	22.6	6.4	0.1	0.0	0.0	0.0
10	3.7	2.0	3.4	2.3	0.77	0.94	0.91	0.93	11.1	4.3	16.2	10.4	0.1	0.1	0.1	0.0
11	4.5	2.4	5.1	1.8	0.77	0.90	0.74	0.93	13.1	4.6	10.9	3.9	0.0	0.0	0.1	0.1
12	5.0	2.7	5.7	2.1	0.77	0.93	0.81	0.93	16.3	7.8	19.8	8.5	0.0	0.1	0.0	0.1
13	5.8	1.6	6.2	1.4	0.84	0.87	0.58	0.91	15.8	8.4	16.0	3.5	0.0	0.1	0.0	0.1
14	4.2	1.6	4.6	2.2	0.78	0.84	0.74	0.95	13.8	6.2	16.8	13.7	0.1	0.0	0.0	0.0
15	4.5	2.7	7.3	3.5	0.70	0.84	0.74	0.95	13.0	8.2	25.3	13.7	0.0	0.1	0.0	0.1
16	3.3	2.3	6.3	2.7	0.86	0.88	0.65	0.96	26.1	11.1	20.0	12.0	0.0	0.1	0.0	0.0
17	2.0	1.0	4.4	2.3	0.78	0.94	0.64	0.91	8.1	3.2	19.7	17.5	0.0	0.0	0.1	0.0
18	2.4	1.5	4.4	1.6	0.78	0.81	0.80	0.93	11.0	3.5	12.9	7.8	0.0	0.0	0.0	0.0
19	3.5	2.0	3.9	2.2	0.73	0.87	0.66	0.90	15.1	5.7	13.2	6.2	0.0	0.1	0.0	0.0
20	4.5	1.6	3.4	2.0	0.86	0.96	0.81	0.93	13.6	2.6	15.8	10.8	0.0	0.0	0.1	0.0
**Mean**	4.3	2.2	5.2	2.7	0.81	0.91	0.77	0.93	15.1	7.1	18.2	10.2	0.0	0.0	0.0	0.0
**Std.**	1.1	0.6	1.3	0.8	0.08	0.05	0.09	0.02	3.7	2.6	3.9	3.5	0.0	0.0	0.0	0.0
